# Fetal Undernutrition Modifies Vascular RAS Balance Enhancing Oxidative Damage and Contributing to Remodeling

**DOI:** 10.3390/ijms23031233

**Published:** 2022-01-22

**Authors:** Maria Sofia Vieira-Rocha, Pilar Rodriguez-Rodriguez, Mariana Ferreira-Duarte, Miguel Faria, Joana Beatriz Sousa, Manuela Morato, Silvia Magdalena Arribas, Carmen Diniz

**Affiliations:** 1 Laboratory of Pharmacology, Department of Drug Science, Faculty of Pharmacy, University of Porto, 4050-313 Porto, Portugal; up201302746@med.up.pt (M.F.-D.); jbsousa@ff.up.pt (J.B.S.); mmorato@ff.up.pt (M.M.); 2 LAQV/REQUIMTE, Faculty of Pharmacy, University of Porto, 4099-002 Porto, Portugal; mfaria@ff.up.pt; 3 Department of Physiology, Faculty of Medicine, Universidad Autonoma de Madrid, 28049 Madrid, Spain; pilar.rodriguezr@uam.es (P.R.-R.); silvia.arribas@uam.es (S.M.A.); 4 Laboratory of Bromatology and Hydrology, Department of Chemical Sciences, Faculty of Pharmacy, University of Porto, 4099-002 Porto, Portugal

**Keywords:** fetal programming of hypertension, renin-angiotensin system, RAS receptors, ACE, ACE2, vascular remodeling, fibrosis, fetal undernutrition

## Abstract

Fetal stress is known to increase susceptibility to cardiometabolic diseases and hypertension in adult age in a process known as fetal programming. This study investigated the relationship between vascular RAS, oxidative damage and remodeling in fetal programming. Six-month old Sprague-Dawley offspring from mothers that were fed ad libitum (CONTROL) or with 50% intake during the second half of gestation (maternal undernutrition, MUN) were used. qPCR or immunohistochemistry were used to obtain the expression of receptors and enzymes. Plasma levels of carbonyls were measured by spectrophotometry. In mesenteric arteries from MUN rats we detected an upregulation of ACE, ACE2, AT_1_ receptors and NADPH oxidase, and lower expression of AT_2_, Mas and MrgD receptors compared to CONTROL. Systolic and diastolic blood pressure and plasma levels of carbonyls were higher in MUN than in CONTROL. Vascular morphology evidenced an increased media/lumen ratio and adventitia/lumen ratio, and more connective tissue in MUN compared to CONTROL. In conclusion, fetal undernutrition indices RAS alterations and oxidative damage which may contribute to the remodeling of mesenteric arteries, and increase the risk of adverse cardiovascular events and hypertension.

## 1. Introduction

The renin-angiotensin system (RAS) is a very complex system involving many peptides and receptors. One of such peptides is angiotensin I (Ang I) that result from angiotensinogen cleavage (by renin) and is transformed into angiotensin II (Ang II) by the angiotensin-converting enzyme (ACE). Ang II acts on specific receptors: angiotensin receptor type I (AT_1_) and type II (AT_2_), with the AT_1_ being the predominant receptor. The classical RAS axis (renin/ACE/Ang II/AT_1_ receptor) is known to promote vasoconstriction and to increase oxidative stress, fibrosis, and inflammation (through activation of AT_1_ receptors). These Ang II-AT_1_ receptor effects are counteracted by a non-classical RAS axis, formed by other peptides, enzymes and receptors [1,2,3]. Among them, the peptide angiotensin-(1–7) (Ang 1–7), that is mainly formed directly from Ang II by angiotensin converting enzyme 2 (ACE2), and the peptide alamandine. These peptides bind, respectively, to the Mas receptor and/or Mas-related G protein-coupled receptor D (MrgD). Among other actions, the ACE2/Ang 1–7 and alamandine axis leads to an increase of nitric oxide (NO) bioavailability and a decrease of oxidative stress, through inhibition of nicotinamide adenine dinucleotide phosphate (NADPH) oxidase and decreased reactive oxygen species (ROS) production [1]. In addition, these non-classical RAS players seem to have a protective role in avoiding remodeling and vascular wall structural alterations [2]. Therefore, unbalance between the classical and non-classical RAS axis—either in the enzymes and subsequent peptide production, or in the expression of the specific receptors—may be implicated in cardiovascular pathologies such as hypertension.

Epidemiological studies indicate an association between exposure to adverse intrauterine environments (e.g., sub-optimal nutrition) and a low birth weight to increased risk of cardiovascular diseases and hypertension, a process known as fetal programming [4,5,6]. Indeed, it has been demonstrated that the fetus can adapt to several adverse intrauterine conditions promoting changes in a variety of physiological processes during fetal development to ensure survival [7].

Experimental data is consistent and supports the idea that RAS-related changes might be a mechanism that underlies hypertension of developmental origins [7,8,9]. In several animal models of the fetal programming of hypertension (FPH), BP rise is controlled by treatment with ACE inhibitors or Ang II receptor antagonists [10,11,12] similar to what occurs in essential hypertension. On the other hand, we have previously shown that in an animal model of FPH induced by maternal undernutrition, cardiac remodeling is related to oxidative stress [13,14], a misbalance in cardiac RAS receptors in early life (rats with 21 days: [15], and facilitation of prejunctional AT_1_ receptors (in vascular sympathetic nerve terminals) mediated by an augmented locally generated Ang II [16]. These data point out at the possible role of vascular RAS in FPH. Given the trophic actions of Ang II, the relationship with oxidative stress and the role of an alteration of the RAS axis in cardiovascular remodeling, we hypothesize that undernutrition during fetal life may induce a disbalance in the vascular RAS axis leading to vascular remodeling and contributing to hypertension. Our aims were to analyze in mesenteric arteries from a rat model of fetal programming induced by maternal undernutrition during gestation (MUN), if there is: (1) changes in the expression of RAS receptors and/or enzymes of the classical and non-classical axis; (2) vascular remodeling, and (3) oxidative damage.

## 2. Results

MUN rats exhibited larger systolic blood pressure (SPB), diastolic blood pressure (DBP) but similar heart rate (HR) when compared to respective data from CONTROL rats (Table 1). Body weight was not different between the two groups of rats in the study.

### 2.1. Influence of Fetal Undernutrition on Arterial ACE and ACE2 Expression and Plasma Ang II Levels

**Figure 1:**
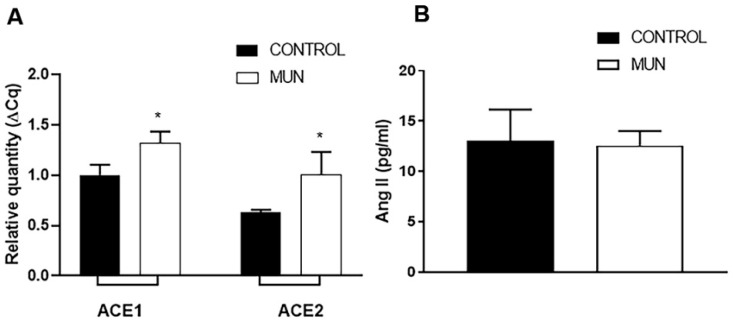
Influence of fetal undernutrition on content of vascular ACE/ACE2 and on plasma Ang II levels in 6-month-old male offspring from rats exposed to maternal undernutrition during pregnancy (MUN) and rats fed *ad libitum* (CONTROL). (**A**) Expression of ACE and ACE2 in mesenteric arteries from MUN and CONTROL rats. RT-PCR analysis of transcripts for ACE/ACE2. Results are normalized to the 28s gene and fold changes between MUN and CONTROL are expressed as mean ± s.e.m., from 6 rats in each group. Significant differences from CONTROL rats: * *p* < 0.05. (**B**) Plasma concentration of Ang II from MUN and CONTROL rats. Values are mean ± s.e.m., from 8 rats in each group. Significant differences from CONTROL rats: * *p* < 0.05.

It was found that the level of expression of RNA for ACE was increased in mesenteric arteries from MUN when compared to CONTROL ones (Figure 1A). Similarly, the level of expression of RNA for ACE2 was also found to be higher in MUN than in CONTROL mesenteric arteries. Despite these differences in vascular tissues, no significant differences in plasma Ang II levels have been detected between MUN and CONTROL rats (Figure 1B).

### 2.2. Influence of Fetal Undernutrition on Renin-Angiotensin System Receptors

**Figure 2:**
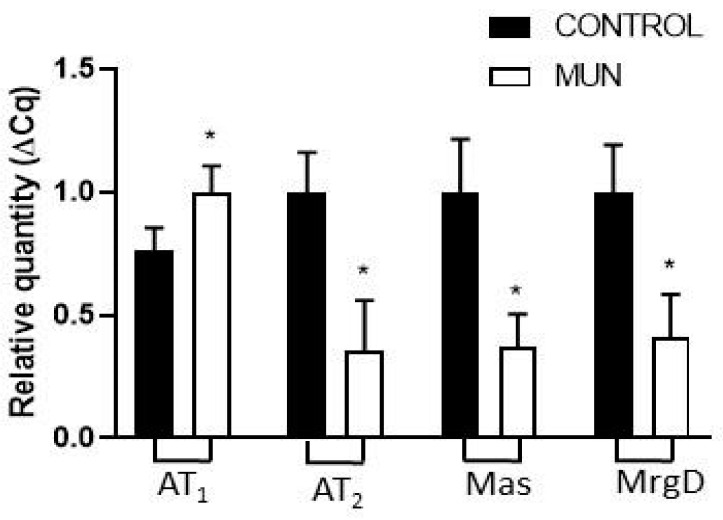
Influence of fetal undernutrition on the expression of RAS receptors in 6-month-old male offspring from rats exposed to maternal undernutrition during pregnancy (MUN) and rats fed *ad libitum* (CONTROL). Expression of AT_1_, AT_2_, Mas and MrgD receptors in mesenteric arteries from MUN and CONTROL rats. RT-PCR analysis of transcripts for AT_1_, AT_2_, Mas and MrgD receptors. Results are normalized to the 28s gene and fold changes between MUN and CONTROL. Values are expressed as mean ± s.e.m. from 6 rats in each group. Significant differences from CONTROL rats: * *p* < 0.05.

The level of expression of RNA for AT_1_ receptors was increased whereas for AT_2_ receptors, Mas receptors and MrgD, RNA expression was reduced in MUN compared to CONTROL mesenteric arteries (Figure 2).

**Figure 3:**
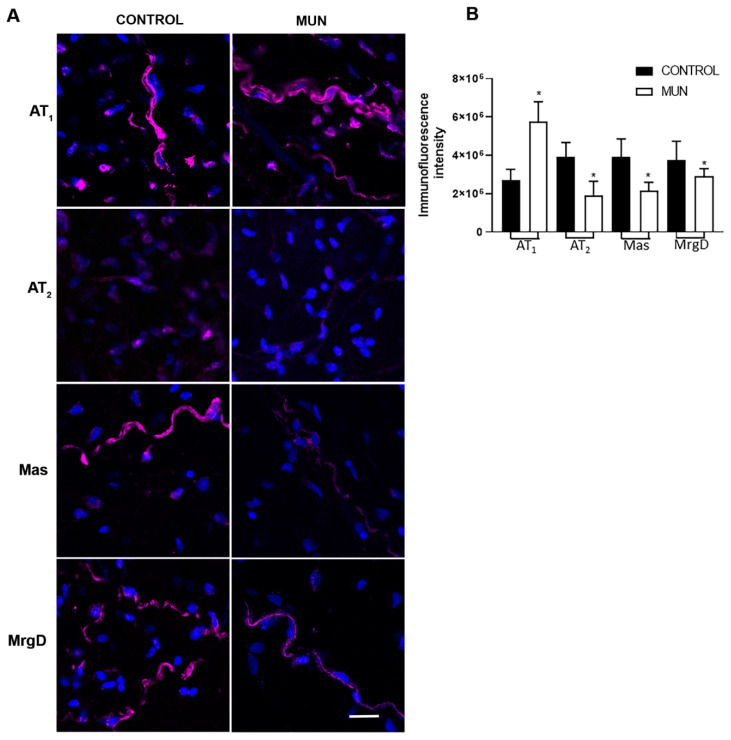
Impact of fetal undernutrition on the distribution profile of RAS receptors in the adventitia layer of mesenteric arteries in 6 -month-old male offspring from rats exposed to maternal undernutrition during pregnancy (MUN) and rats fed *ad libitum* (CONTROL). (**A**) Laser scanning confocal microscopy representative images of CONTROL and MUN mesenteric arteries exhibiting AT_1_, AT_2_, Mas or MrgD receptors (red) and nuclei (blue); (**B**) and quantitative analysis of LSCM images. Values are expressed as mean ± s.e.m. from 6 rats in each group. Significant differences from CONTROL rats: * *p* < 0.05.

Evidence over the past decade has demonstrated that wall changes in the adventitia occur early in the course of vascular disease development, before changes are seen in the intima or the media [17]. Thus, we observed the distribution profile of RAS receptors in the adventitia mesenteric artery of CONTROL and MUN animals by immunohistochemistry (Figure 3A), in order to determine if fetal undernutrition modifies RAS receptors’ distribution profile or expression. Data revealed that all RAS receptors, AT_1_, AT_2_, Mas and MrgD, were present, evidencing a differential distribution profile. A higher immunoreactivity for AT_1_ receptors was detected, while lower immunoreactivities for AT_2_, Mas and MrgD receptors were observed in mesenteric arteries from MUN rats compared to CONTROL rats (Figure 3A). Histomorphometric analysis corroborates that fetal undernutrition increases the immunoreactivity for AT_1_ receptors and decreases the immunoreactivity detected for the other RAS receptors in MUN mesenteric arteries (Figure 3B).

### 2.3. Influence of Fetal Undernutrition on NADPH Oxidase and Oxidative Damage

**Figure 4:**
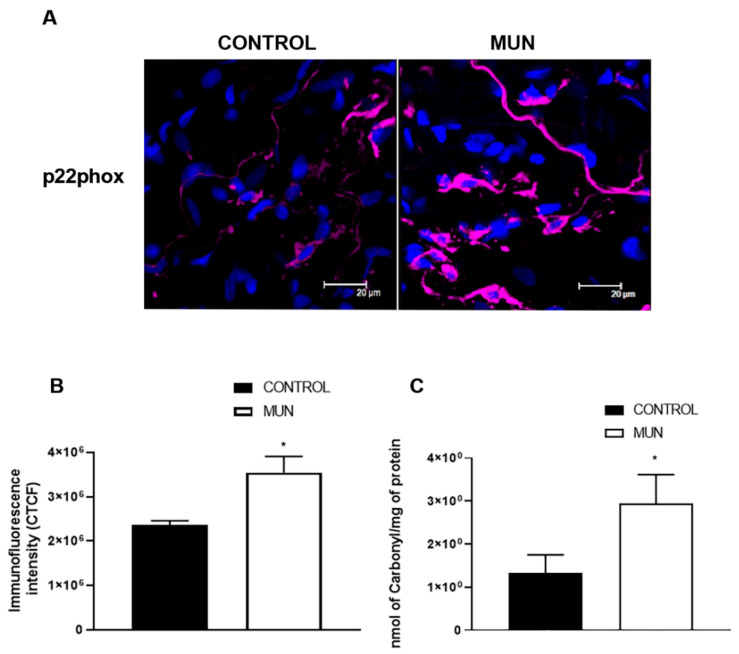
Influence of fetal undernutrition on NADPH oxidase and oxidative stress in 6-month-old male offspring from rats exposed to maternal undernutrition during pregnancy (MUN) and rats fed *ad libitum* (CONTROL). (**A**) Laser scanning confocal microscopy representative images of CONTROL and MUN mesenteric arteries exhibiting p22phox (red) and the nuclei (blue); (**B**) quantitative analysis of LSCM images. (**C**) Plasma oxidative status (levels of Carbonyls). Values are mean ± s.e.m. from 6 rats in each group. Significant differences from CONTROL rats: * *p* < 0.05.

Since NADPH oxidase is the main source of ROS in vascular tissues [18,19], the level of expression of NADPH oxidase was analyzed. Immunohistochemistry was used to determine the expression of a subunit (p22phox) of the enzyme NADPH oxidase. A higher immunoreactivity for p22phox was observed in mesenteric arteries from MUN rats when compared to CONTROL (Figure 4A,B). Carbonyl levels, representative of oxidative damage to proteins, were significantly elevated in plasma from MUN compared to CONTROL rats (Figure 4C).

### 2.4. Influence of Fetal Undernutrition on Vascular Wall Remodelling and BP

**Figure 5:**
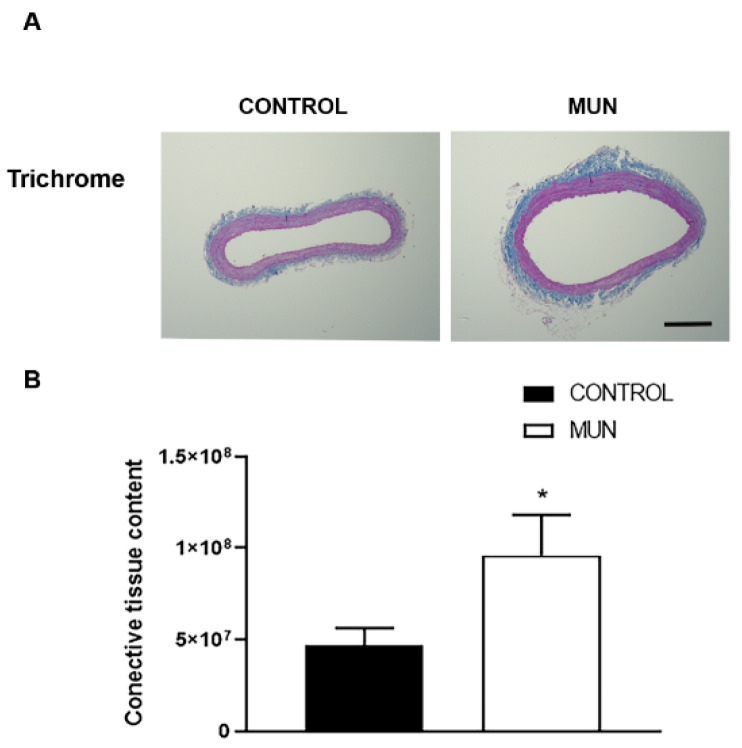
Influence of fetal undernutrition on the perivascular content of connective tissue in 6-month-old male offspring from rats exposed to maternal undernutrition during pregnancy (MUN) and rats fed *ad libitum* (CONTROL). (**A**) Representative images of mesenteric arteries from CONTROL and MUN. (**B**) Quantification data on the content of connective tissue. Results are expressed as a percentage of the total artery area. Images were obtained from Masson’s trichrome stained arteries (scare bar = 300 μm). Values are mean ± s.e.m. from 6 rats in each group. Significant differences from the respective control rat: * *p* < 0.05.

**Figure 6:**
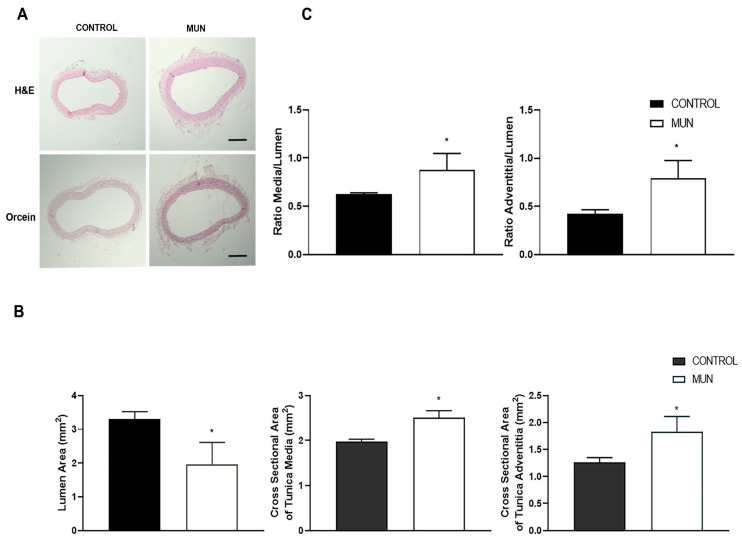
Influence of fetal undernutrition on mesenteric artery morphology in 6-month-old male offspring from rats exposed to maternal undernutrition during pregnancy (MUN) and rats fed *ad libitum* (CONTROL). Representative images (**A**) obtained from hematoxylin-eosin (scare bar = 300 μm) and orcein (scare bar = 500 μm) stained mesenteric arteries from CONTROL and MUN; the graphics show (**B**) the area of lumen, of tunica media and of tunica adventitia; (**C**) the ratio media/lumen and the ratio adventitia/lumen. Values are mean ± s.e.m. from 6 rats in each group. Significant differences from the respective control rat: * *p* < 0.05.

RAS receptor types differentially influenced cellular proliferation, inflammation, oxidative stress and fibrosis, with AT_1_ receptors increasing these processes while the other RAS receptors are known to have opposite actions, leading to putative changes in vascular morphology. In addition, ROS (generated by enzymes such as NADPH oxidase) and oxidative damage can also modify vascular morphology. Thus, in MUN and CONTROL histological sections of mesenteric arteries, vascular morphology was evaluated (Figure 5 and Figure 6). Data evidenced a significant increase in connective tissue content observed in MUN arteries compared to that from CONTROL (Figure 5).

Data also revealed significantly larger media and adventitia thickness, as well as cross sectional areas of both layers, and a lower lumen diameter in MUN arteries comparatively to that obtained in CONTROL (Figure 6A,B). Moreover, ratios of media/lumen and of adventitia/lumen were significantly increased in MUN relative to those from CONTROL group (Figure 6C).

## 3. Discussion

The current study shows that fetal undernutrition induces alterations in the arterial balance between classic and counter-regulatory arms of the RAS in rat adulthood (6 months after birth) which can justify, at least in part, the vascular remodeling and fibrosis. In addition, this study highlights the arterial adventitia as an important player associated with this pathophysiological alteration.

Our data evidenced an upregulation of both ACE and ACE2 in the mesenteric arteries from MUN rats. Elevated ACE2 can be interpreted as a compensatory mechanism in response to an increase in Ang II levels, by raising its degradation into Ang 1–7, as previously proposed [8]. In fact, in young MUN rats, we have evidenced increased Ang II plasma levels [15]. In addition, previous work provided evidence that increased vascular ACE leads to increased local Ang II formation [20,21] and the vascular expression of ACE and ACE2 enzymes have been proposed as indicators of local Ang II levels [20,22]. Usually, lower levels of ACE2 expression are found in vascular smooth muscle cells (VSMC) and in the adventitia of large blood vessels [23]. This was also observed in the current study in mesenteric arteries from CONTROL rats. This is also evidenced by the fact that the circulating levels of ACE2, which are typically low or even undetectable in physiological conditions, are also increased in experimental conditions of diabetic hypertension [24].

In the current study in adult rats, no significant differences in plasma Ang II levels were detected between MUN and CONTROL rats. Such evidence is corroborated by other authors that have found that, in a maternal GC-exposed sheep model, circulating levels of Ang II in 6-month-old offspring were not significantly changed [9]. By contrast, Yu et al. reported that plasma levels of Ang II were reduced in male adult offspring of GC-treated pregnant rats [25]. Although this might contradict our vascular data, it is known that the plasma Ang II levels do not reflect local Ang II levels, which have more relevant pathophysiological effects [26].

We also studied how maternal undernutrition affects the expression of RAS receptors in the vasculature in adult age and observed a higher expression of AT_1_ receptors in MUN mesenteric arteries when compared to CONTROL rats. This was corroborated by LSCM images which show higher immunoreactivity for AT_1_ receptors in the adventitia. Upregulation of AT_1_ receptors is also in line with other works in kidneys from maternal undernutrition of rat offspring [27], and also in the brain of adult rats exposed to low protein diet [28,29] or to nicotine [30] during fetal development. Similarly, increased pulmonary AT_1_ receptor expression was found in lungs of mice exposed to hypoxia [31] and in the kidney of near-term ovine fetuses exposed to high altitude [32]. We have also observed larger AT_1_ receptors in intramyocardial arteries from MUN rats in young age [15].

Our previous data adds support to the implication of alterations in AT_1_ receptors in FPH, through functional alterations. The relationship between RAS and the sympathetic nervous system has been addressed, particularly in the adventitia in a previous work of our group, where it was demonstrated that the release of noradrenaline from sympathetic nerve terminals is under the influence of Ang II [33]. We demonstrated that Ang II is paracrine factor on presynaptic AT_1_ receptors, facilitating sympathetic neurotransmission, and this tonic facilitation is increased in MUN vessels [16], which could contribute to enhanced vasoconstriction and blood pressure.

Besides, a lower expression/immunoreactivity of AT2 receptor was observed in the mesenteric artery of MUN rats, which is in line with data previously reported in the kidney of offspring exposed to maternal undernutrition and low protein diet [34,35], hypoxia [32], GC [36] and our previous report on intramyocardial arteries [15]. Additionally, a lower expression/immunoreactivity for Mas and MrgD receptors were also observed in mesenteric arteries from MUN rats. Downregulation of Mas receptors were reported in the brain of a 6-month sheep exposed to maternal GC [37]. The same group demonstrated that the reduced expression of Mas receptors and increased metabolism of Ang 1–7 in the brain may have contributed to the loss of Ang 1–7 induced tone, as well as the enhanced responsiveness of the Ang II-AT1 receptor pathway in GC-dependent programming. The present results support similar findings in the vascular wall from MUN rats. We were not able to measure Ang 1–7 levels, which would have been interesting. Based on the larger expression level of ACE2 in MUN rats, an increase would be expected. However, the potential beneficial effects would not be achieved, due to the reduction in receptor expression. On the other hand, it is possible that a potential increase in Ang 1–7 could eventually contribute to the downregulation of the Mas receptor.

The present data supports the role of local alterations in RAS axis in the vascular remodeling of MUN rats. Our group has previously demonstrated that the presence of AT1 and AT2 receptors in Schwann cells, that have a role on noradrenaline neurotransmission [38,39], support a trophic role of these cells and of its close association with neurons. These data suggest that enhanced levels of Ang II (as a consequence of increased levels of ACE) may be exerting trophic actions through AT1 receptors, contributing to the hypertrophy of sympathetic innervation observed in MUN rats (data not shown). Images also reveal that these receptors are present in other adventitia cells, possibly macrophages (lobulated nuclei), mesenchymal cells or fibroblasts (oval nuclei). Activation of fibroblasts may be responsible for proliferation and an increased amount of connective tissue in MUN mesenteric arteries. Indeed, the adventitia has been identified as an important source of ROS that may function as paracrine molecules modulating neighbouring cells and, thus, contributing to vasoconstriction and vascular remodeling [40]. Adventitial fibroblasts have been shown to produce substantial amounts of NADPH oxidase-derived ROS in response to vascular injury or vasoactive substances, and play an active role in collagen deposition and vascular remodeling [41]. We have gathered previous evidence on increased collagen, associated with elevation of AT1 receptors in intramyocardial arteries from MUN rats [15]. Among them, Ang II is recognized as one of the key humoral factors implicated in NADPH oxidase activation in the vascular wall [42,43,44]. The present data also supports this mechanism in MUN rat mesenteric arteries by showing a higher immunoreactivity to p22phox, alongside the stabilizing subunit of NADPH oxidase, which is the main enzymatic system responsible for superoxide anion in the vasculature [42,45]. We have evidence that enhanced protein expression of this NADPH oxidase subunit is in the heart of MUN rats [13]. In our previous study and in the present study, we detected higher levels of carbonyls in plasma, which is a marker of oxidative damage to proteins.

Our finding that this subunit is increased suggests that superoxide anion is also locally increased. This is in line with previous studies that indicated an association between plasma oxidative damage biomarkers (protein carbonyls), superoxide anion production (evaluated by DHE) and NADPH activity [46]. Some studies demonstrate that adventitia is the most complex compartment of the vessel that integrates key regulators of vessel wall function [40,47] and data evidence that wall changes in the adventitia occur early in the course of vascular disease development before changes are seen in the intima or the media [17]. In the literature, the influence of intima to media layers and vice versa is well established: endothelial cells can release mediators that alter the tonus of VSMC, and VSMC can also influence the activity of endothelial cells [48]. Recent data has also highlighted the interplay of intima with adventitia since endothelial cells have been shown to release mediators able to alter vascular sympathetic tonus [39,48,49].

An increased level of ROS, if not counteracted by enough antioxidants, may lead to oxidative damage. In fact, in the MUN model of FPH, our group has previously demonstrated that at the age of 21 days, MUN rats exhibit lower levels of plasmatic antioxidants [50]. Higher expressions of NADPH oxidase and Xanthine oxidase have also been reported in the heart of these animals [13]. The presence of higher NADPH oxidase in the vasculature of this model of FPH is suggestive of increased ROS generation [14] that can contribute to hypertension in various ways as through remodeling and by promoting endothelial dysfunction [51,52,53]. In fact, the remodeling of mesenteric vessels was previously associated with increased levels of ROS (measured by dihydroethidium (DHE) in the adventitia [54]. Moreover, previous data suggested that superoxide anion release from the adventitia might reduce endothelial NO bioavailability [48,55], which would also enhance vasoconstriction and increase resistance. Interestingly, it has been demonstrated that an increased ROS is a primary mechanism whereby Ang II causes an increased sympathetic nervous system activity in a complex mechanism involving the contribution of the adaptive immune response [56]. Taken together, present data and data from our previous study [16] indicate that increased Ang II/AT_1_ receptor axis in the adventitia may be implicated in the development of vascular remodeling, fibrosis and enhanced sympathetic neurotransmission in MUN rats, being ROS possible mediators. Moreover, Ang II and noradrenaline are well-known trophic factors and their elevation can favor the hypertrophy of VSMC [57,58].

The adventitia has not been well explored in the context of FPH, and, to our best knowledge, this is the first study that explores the adventitia role in FPH, demonstrating that mesenteric arteries of rats exposed to undernutrition in fetal life have significantly increased adventitia area and connective tissue content. Furthermore, the presence of an increased media–lumen ratio and adventitia–lumen ratio in MUN was also demonstrated. Since wall–lumen is associated with higher risk of adverse cardiovascular events [59,60], the remodeling process in MUN does not seem to be adaptive, and is likely to contribute to cardiovascular damage.

In response to vascular stress or injury, adventitial cells are often the first to be activated and re-programmed to then influence the tone and structure of the vessel wall, to initiate and perpetuate chronic vascular inflammation, and to act and stimulate the expansion of the vasa vasorum, which can act as a conduit for continued inflammatory and progenitor for cell delivery to the vessel wall [40]. Under conditions of elevated BP, the adventitia becomes the predominant wall component due to the profound influence on intima and media function in disease [40,61]. As so, our work corroborates others’ findings and demonstrates the importance of adventitia, suggesting a modulatory role on vascular function [48].

The current study is also in line with epidemiological studies indicating an association between exposure to adverse perinatal environments (e.g., sub-optimal nutrition) and elevated blood pressure and/or vascular dysfunction in later life. In fact, the contribution of the altered angiotensinogen gene, ACE gene, or AT_1_ receptor gene was previously ruled out [62] in a study that analyzed 2.4 million single nucleotide polymorphisms (SNPs) in approximately 300,000 patients with essential hypertension worldwide.

## 4. Materials and Methods

### 4.1. Chemicals

The following drugs were used: entellan (mounting medium), hematoxylin-eosin, Masson’s trichrome, and orcein purchased from Merck (Darmstadt, Germany). The following antibodies were used: rabbit polyclonal anti-AT_1_ receptor (Santa Cruz Biotechnology, Inc., Dallas, TX, USA); rabbit polyclonal anti-AT_2_ receptor (Santa Cruz Biotechnology, Inc.); rabbit polyclonal anti-Mas (Alomone labs, Jerusalem, Israel); rabbit polyclonal anti-MrgD (Alomone labs); goat polyclonal anti-p22phox (Santa Cruz Biotechnology, Inc.); Alexa Fluor^®^ 594 anti-goat IgG (H+L) antibody, highly cross-adsorbed; and Alexa Fluor^®^647 anti-rabbit IgG (H+L) antibody, highly cross-adsorbed (Invitrogen, Life Technologies, SA, Madrid, Spain). A vectashield mounting medium with DAPI (Vector Laboratories, Peterborough, UK) was used. The RNAlater^®^-ICE and TRIzol were from Invitrogen (Thermo Fisher Scientific, Waltham, MA, USA) An Xpert cDNA Synthesis Mastermix kit (GRiSP, Porto, Portugal) and SsoAdvanced Universal SYBR Green Supermix (Bio-Rad, Milan, Italy) were also used. The primers used are shown in Table 2.

Stock solutions were made up in ultrapure water and diluted in superfusion medium immediately before use.

### 4.2. Experimental Model of FPH

Sprague–Dawley rats were maintained at the animal house facility of the Universidad Autónoma de Madrid. All experimental procedures were approved by the Ethics Review Board of Universidad Autónoma de Madrid CEI-UAM 96-1776-A286) and Comunidad Autónoma de Madrid PROEX 04/19) according to the Guidelines for the Care and Use of Laboratory Animals (National Institutes of Health publication no. 85-23, revised in 1996), the Spanish legislation (RD 1201/2005) and the Directive 2010/63/EU on the protection of animals used for scientific purposes.

The rats were housed in buckets 36.5/21.5/18.5 cm (length/width/height) on aspen wood bedding, under controlled conditions of 22 °C, 40% relative humidity and 12–12 light–dark photoperiod. The Animal Health Monitoring indicated that they were free from pathogens that may interact with any of the parameters studied. The ARRIVE Guidelines were followed for reporting in vivo experiments [63].

FPH model based on global maternal nutrient restriction was induced as previously described [50,64]. Two study groups were established: a CONTROL group, with ad libitum feeding throughout pregnancy and lactation, and a group with intake restriction during part of gestation. This last group of rats had ad libitum diet during the first half of the gestation (from day 1 to 10) and then they were fed with 50% of the intake of a pregnant rat (from day 11 until delivery). The maximum daily intake of rat chow was previously determined in a group of pregnant rats as 24 g/day. After delivery and through the lactation period, the mothers were fed ad libitum. The mothers were fed with a breeding diet (Euro Rodent Diet 22; 5LF5, Labdiet, Madrid, Spain) containing 55% carbohydrates, 22% protein, 4.4% fat, 4.1% fiber, and 5.4% mineral and 12.2% humidity. Drinking water was provided ad libitum to all animals. Immediately after birth, the offspring were weighed individually and sexed, and the litter was randomly standardized to 12 rats, 6 males and 6 females, if possible. The rest of the litter was sacrificed with CO_2_. At the age of 6 months, the male offspring from the two experimental groups were analyzed: those from rats exposed to maternal undernutrition during pregnancy (MUN) and those from control rats with mothers fed ad libitum during pregnancy (CONTROL). Rats from at least three different litters were used for each experimental protocol.

The animals were first weighed and then anesthetized to measure the haemodynamic parameters (see below). Finally, they were sacrificed by exsanguination, where blood was collected alongside the mesenteric arteries, as well as other tissues for additional studies following the 3Rs.

The mesenteric arteries were washed and immediately used.

### 4.3. Hemodynamic Parameters Measurement and Blood Sample Collection

Hemodynamic parameters were determined as previously described [13]. Briefly, systolic blood pressure (SBP), diastolic blood pressure (DBP) and heart rate (HR) were measured in anesthetized animals (37.5 mg/kg ketamine hydrochloride and 0.25 mg/kg medetomidine hydrochloride i.p.). Following this, after the anesthesia, a cannula was inserted into the right iliac artery and connected to a pressure transducer (Statham, Harvard Apparatus GmbH, Germany). The wave blood pressure was recorded on a PC computer, using the data acquisition with the PowerLab system (ADInstruments) for 60 min. SBP, DBP and HR were measured in the final portion of the recorded data. At the end of the recording period, a blood sample was obtained by performing cardiac puncture into EDTA or heparin-containing tubes to measure Ang II or carbonyl levels, respectively. Thereafter, the blood was centrifuged (3000 rpm, 15 min, 4 °C) and the supernatant plasma was stored at −80 °C until used.

### 4.4. Plasma Measurements

Plasma protein carbonyls were assessed with a 2,4-dinitrophenylhydrazine-based assay [65], as previously described [50]. The protein carbonyl concentration was determined using extinction coefficient of 2,4-dinitrophenylhydrazine (ε = 22,000 M/cm) and expressed as nmol/mg protein. Protein content was determined by a Coomassie blue– based microtiter plate assay according to manufacturer instructions (Bio-Rad). The absorbance was measured at 595 nm in a Synergy HT Multi-Mode Microplate Reader (Bio-tek).

Ang II was first extracted from plasma samples by solid phase extraction (SPE) (Discovery^®^DSC-Ph SPE Tube, Supelco, PA, USA), as previously described [66]. Briefly, the columns were activated with a sequence of 5 mL of the following solvents in this order: methanol, tetrahydrofuran, hexane, methanol and ultrapure water. Thereafter, plasma samples were placed in the columns and the extraction was made with another sequence of 5 mL of the following solvents: ultrapure water, acetic acid 4% and finally a mixture consisting in ethanol–acetic acid–water (90:4:6) (prepared on time from a cold stock solution). The last 5 mL of the mixture was collected, evaporated with nitrogen, followed by lyophilization, and kept frozen at −80 °C until needed. Ang II levels were quantified by a peptide enzyme immunoassay (EIA) (Peninsula Laboratories International, Inc., San Carlos, CA, USA) according to the manufacturer’s instructions. Briefly, in a 96-well plate, each sample and standard were loaded in duplicate, and, after reaction, absorbance was read at 450 nm on a plate reader (Synergy HT MultiMode Microplate Reader, Biotek, VT, USA) within 10 min after the end of the protocol. The concentration of Ang II in the sample is inversely proportional to the measured optical density.

### 4.5. Immunohistochemistry

Immunohistochemistry procedures were previously described [67]. Briefly, four arterial segments were obtained from each mesenteric artery and immediately placed in cold phosphate buffer solution (PBS; in g/L): NaCl 8.0, Na_2_HPO_4_.2H_2_O 0.77, KCl 0.20, KH_2_PO_4_ 0.19 (pH 7.2). Each segment was longitudinally opened and fixed (paraformaldehyde 4% PBS; 50 min; room temperature). After two 15 min PBS washing cycles, artery segments were incubated with the primary antibodies in the study (rabbit polyclonal antibodies against angiotensin receptors subtypes, anti-AT_1_, anti-AT_2_, anti-Mas, anti-MrgD- 1:100 dilution at 4 °C overnight- and goat polyclonal anti-p22phox to stain NADPH oxidase-1:200 dilution, overnight, 4 °C). Thereafter, tissues were incubated with the Alexa 647 anti-rabbit fluorescent secondary antibody (1:1000 dilution, 1 h, room temperature) or with the Alexa 594 anti-goat fluorescent secondary antibody (1:1000 dilution, 1 h, room temperature). Negative controls were incubated on adjacent sections using 10% normal horse serum or blocking solution instead of the primary antibody. After three PBS washing cycles, the arteries were mounted intact with an anti-fading agent (Vectashield mounting medium with DAPI).

Artery segments were visualized with a Leica SP2 Laser scanning confocal microscopy (LSCM) system (Leica Microsystems, Wetzlar, Germany) fitted with an inverted microscope (×63 oil immersion lens). Stacks of 1-μm-thick serial optical images were captured from five randomly chosen regions along the adventitial layer of the mesenteric artery, which was identified by the shape and orientation of the nuclei stained with DAPI [68]. The adventitia was scanned along each mesenteric artery and the resulting images were reconstructed separately for each wavelength. Two stacks of images were sequentially obtained at different wavelengths: the first stack was taken with the Ex 405 nm and Em 412–470 nm wavelength to visualize cell nuclei (DAPI staining). The second was taken with the Ex 633 nm and Em 640–720 nm wavelength to detect RAS receptors’ distribution or NADPH oxidase, stained with the secondary antibody Alexa Fluor 647 (different subtypes depending on primary antibody). Image acquisition was always performed under the same laser power, brightness, and contrast conditions. The resulting images were reconstructed separately for each wavelength for later quantification.

Quantitative analysis of confocal z-stacks images was performed using image analysis software (PAQI, CEMUP, Porto, Portugal) as previously [69]. Briefly, a sequential routine was designed and developed to analyze each fluorescent signal used. PAQI software measured the surface area and strength of the fluorescence signal marking the receptors or the p22phox.

### 4.6. qPCR

Mesenteric arteries were dissected ensuring maximum protection of the RNA and were stored in sterile tubes at −80 °C until their use. RNAlater^®^-ICE was used to defrost and process the samples. RNA was extracted using TRIzol and a Precelys homogenizer (Bertin Technologies, France) following the manufacturer’s instructions. The total RNA integrity was evaluated on 1% agarose and its purity and concentration were assessed spectrophotometrically (Synergy HT MultiMode Microplate Reader, Biotek, VT, USA) at 260 nm and 280 nm. A quantity of 1 μg of total RNA was reverse transcribed with Xpert cDNA Synthesis Mastermix Kit (GRISP, Porto, Portugal). Real-time PCR reactions were carried out in a 96-well CFX RT-PCR System (Bio-Rad, Milan, Italy). Each reaction was performed using 10 μL, including 2 ng of template cDNA, 0.4 μM of each primer and 1X SsoAdvanced Universal SYBR Green Supermix (Bio-Rad). Gene amplification protocol started with 95 °C for 30 s, followed by 39 cycles at 95 °C for 15 s and 60 °C for 30 s. Each assay was performed in a triplicate with a negative control. The relative expression of the genes Renin, ACE, ACE2, AT1 receptor, AT2 receptor, Mas receptor and MrgD [70] were calculated using the 2−∆∆Ct method [71], normalized to the expression of three housekeeping genes: HPRT-1: hypoxanthine phosphoribosyltransferase I; Hmbs: Hydroxymethylbilane synthase; and Papbn-1: Polyadenylate-binding protein 1 [72].

### 4.7. Histology

Serial 2 µm thickness sections of mesenteric arteries, previously fixed in paraformaldehyde 4% PBS, were dewaxed in xylene before being hydrated in decreasing concentrations of alcohols and stained with haematoxylin-eosin using Masson’s trichrome to assess connective tissue components and orcein and to detect elastic fibers. Each tissue was cut in five levels along the length of the vessel to ensure data represented the putative mesenteric artery heterogeneity rather than only a specific location of the artery. Each batch represented histochemical staining for one type of dye and included sections from all five levels of mesenteric arteries from each animal group. This procedure was repeated three times to create three batches. In total, for each treatment, 150 sections were obtained. Sections were stained using each dye and divided according to animal source. Within each of these groups, a random selection of the sections was carried out.

Stained sections were visualized using a high-resolution Zeiss Axiocam 105 color digital camera mounted on a Zeiss Primo Star microscope, using an X10 objective, to analyze the arterial lumen, media, and adventitia layer. Morphometry was performed with Image J software [73], and data of lumen diameter, and a cross-sectional area of the media, the adventitia and collagen content were obtained.

### 4.8. Statistics

Statistical analyses were performed with Graph-Pad Prism (version 8.3). Sample size was calculated assuming a probability error of alpha type of 5% (*p* < 0.05) and potency of 80%. The normality of the variables was evaluated with the Kolmogorov–Smirnov test. Since the variables followed a normal distribution, parametric tests were used. Results were expressed as mean ± s.e.m. Differences of means were compared using one- or two-way ANOVA, followed by post-hoc Holm–Sidak’s multi-comparison *t* test or Student’s *t* test. A *p* value lower than 0.05 was considered to denote statistically significant differences.

## 5. Conclusions

**Figure 7:**
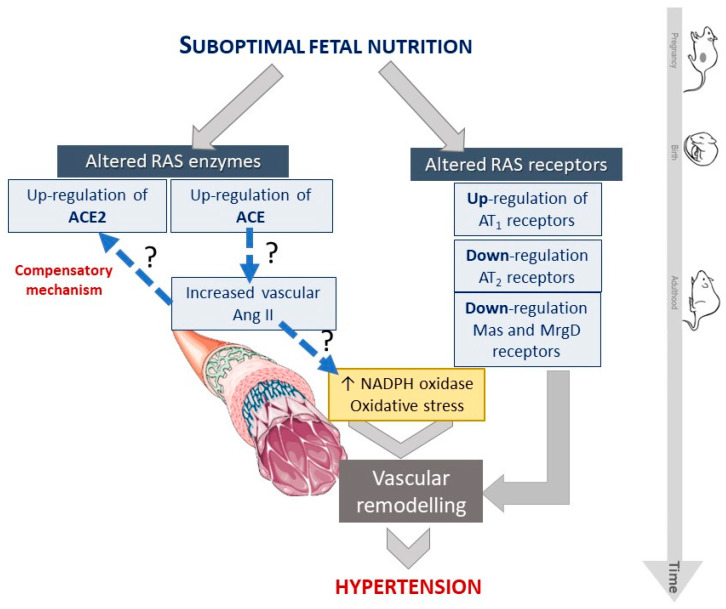
Scheme representing the impact of RAS and NADPH oxidase alterations induced by fetal undernutrition that led to vascular remodeling/fibrosis and hypertension.

Overall, our data (Figure 7) suggests that maternal undernutrition induces changes in the content of RAS components.

The upregulation of ACE and ACE2 enzymes, and of AT_1_ receptors, among a downregulation of AT_2_, Mas and MrgD receptors, have an impact in NADPH oxidase expression, and thus, influence the redox status and contribute to vascular remodeling and fibrosis associated with hypertension.

## Figures and Tables

**Table 1:** Influence of fetal undernutrition on the haemodynamic parameters and body weight.

	Body Weight (g)	Systolic Pressure (mmHg)	Diastolic Pressure (mmHg)	Heart Rate (BPM)	N
CONTROL (n = 7)	486.6 ± 18.6	125.6 ± 5.1	68.7 ± 4.2	258 ± 8	6
MUN (n = 7)	460.18 ± 8.1	151.0 ± 3.9 *	90.3 ± 4.6 *	254 ± 10	6

Six-month-old male offspring from rats exposed to maternal undernutrition during pregnancy (MUN) and rats fed *ad libitum* (CONTROL). Significant differences from CONTROL rats: * *p* < 0.05.

**Table 2:** Reference and target genes: primer specifications.

Gene	Primer Sequence (5′→3′)	Length (bp)	GenBank No.
**Ren**	F: CTAAAGCATCTCGCCAAGG		AB188298
	R: ACCAGGGATGTGTCGAATGA		
**Ace**	F: TCCTATTCCCGCTCATCT	127	NM_012544.1
	R: CCAGCCCTTCTGTACCATT		
**Ace 2**	F: GAATGCGACCATCAAGCG	228	AY881244
	R: CAAGCCCAGAGCCTACGA		
**Agtr1**	F: TCTGGATAAATCACACAACCCTC	77	NM_030985.4
	R: GAGTTGGTCTCAGACACTATTCG		
**Agtr2**	F: CTGGCAAGCATCTTATGTAGTTC	115	U22663.1
	R: ACAAGCATTCACACCTAAGTATTC		
**Mas1**	F: ACTGTCGGGCGGTCATCATC	272	NM_012757.2
	R: GGTGGAGAAAAGCAAGGAGA		
**Mrgprd**	F: CAGCCTCGGCGGCTCTA		293648
	R: CCAACGGCAGAGAACAGGTAAG		
**Hprt-1**	F: CCCAGCGTCGTGATTAGTGATG	110	NM_012583
	R: TTCAGTCCTGTCCATAATCAGTCC		
**Hmbs**	F: TCTAGATGGCTCAGATAGCATGCA	76	NM_013168
	R: TGGACCATCTTCTTGCTGAACA		
**Papbn-1**	F: TATGGTGCGACAGCAGAAGA	110	116697
	R: TATGCAAACCCTTTGGGATG		

Primers selected for renin-angiotensin study, including the main enzymes (renin, ACE, ACE2) and receptors (AT1, AT2, Mas and MrgD). The 3 housekeeping genes used were: Hprt-1, Hmbs and Papbn-1. F, forward primer sequence (5′–3′); R, reverse primer sequence (5′–3′).

## Data Availability

The data presented in this study are available on request from the corresponding author (M.S.V.R.).

## References

[1] Santos R.A., Ferreira A.J., Verano-Braga T., Bader M. (2013). Angiotensin-converting enzyme 2, angiotensin-(1-7) and Mas: New players of the renin-angiotensin system. J. Endocrinol..

[2] Etelvino G.M., Peluso A., Santos R.A.S. (2014). New Components of the Renin-Angiotensin System: Alamandine and the Mas-Related G Protein-Coupled Receptor D. Curr. Hypertens. Rep..

[3] Villela D.C., Passos-Silva D.G., Santos R.A. (2014). Alamandine: A new member of the angiotensin family. Curr. Opin. Nephrol. Hypertens..

[4] Barker D.J., Osmond C. (1988). Low birth weight and hypertension. BMJ.

[5] Barker D.J., Clark P.M. (1997). Fetal undernutrition and disease in later life. Rev. Reprod..

[6] Alexander B.T. (2006). Fetal programming of hypertension. Am. J. Physiol. Regul. Integr. Comp. Physiol..

[7] Alexander B.T., Dasinger J.H., Intapad S. (2015). Fetal Programming and Cardiovascular Pathology. Compr. Physiol..

[8] Chappell M.C., Marshall A.A., Alzayadneh E.M., Shaltout H.A., Diz D.I. (2014). Update on the Angiotensin converting enzyme 2-Angiotensin (1-7)-MAS receptor axis: Fetal programing, sex differences, and intracellular pathways. Front. Endocrinol..

[9] South A.M., Shaltout H., Washburn L.K., Hendricks A.S., Diz D.I., Chappell M.C. (2019). Fetal programming and the angiotensin-(1-7) axis: A review of the experimental and clinical data. Clin. Sci..

[10] Manning J., Vehaskari V.M. (2005). Postnatal modulation of prenatally programmed hypertension by dietary Na and ACE inhibition. Am. J. Physiol. Integr. Comp. Physiol..

[11] Langley-Evans S.C., Jackson A.A. (1995). Captopril normalises systolic blood pressure in rats with hypertension induced by fetal exposure to maternal low protein diets. Comp. Biochem. Physiol. Part A Physiol..

[12] Sherman R.C., Langley-Evans S.C. (2000). Antihypertensive treatment in early postnatal life modulates prenatal dietary influences upon blood pressure in the rat. Clin. Sci..

[13] Rodríguez-Rodríguez P., De Pablo L.L., García-Prieto C.F., Somoza B., Quintana-Villamandos B., De Diego J.J.G., Gutierrez-Arzapalo P.Y., Ramiro-Cortijo D., Gonzalez-Garcia M.C., Arribas S.M. (2017). Long term effects of fetal undernutrition on rat heart. Role of hypertension and oxidative stress. PLoS ONE.

[14] Rodríguez-Rodríguez P., Cortijo D.R., Reyes-Hernández C.G., De Pablo A.L.L., González M.C., Arribas S.M. (2018). Implication of Oxidative Stress in Fetal Programming of Cardiovascular Disease. Front. Physiol..

[15] Rodríguez-Rodríguez P., Vieira-Rocha M., Quintana-Villamandos B., Monedero-Cobeta I., Prachaney P., de Pablo A.L., González M., Morato M., Diniz C., Arribas S. (2021). Implication of RAS in Postnatal Cardiac Remodeling, Fibrosis and Dysfunction Induced by Fetal Undernutrition. Pathophysiology.

[16] Vieira-Rocha M., Rodríguez-Rodríguez P., Sousa J., González M., Arribas S., de Pablo A.L., Diniz C. (2018). Vascular angiotensin AT1 receptor neuromodulation in fetal programming of hypertension. Vasc. Pharmacol..

[17] Havelka G.E., Kibbe M.R. (2011). The Vascular Adventitia: Its Role in the Arterial Injury Response. Vasc. Endovasc. Surg..

[18] Berry C., Hamilton C.A., Brosnan M.J., Magill F.G., Berg G.A., McMurray J.J., Dominiczak A.F. (2000). Investigation into the sources of superoxide in human blood vessels: Angiotensin II increases superoxide production in human internal mammary arteries. Circulation.

[19] Sirker A., Zhang M., Murdoch C., Shah A.M. (2007). Involvement of NADPH Oxidases in Cardiac Remodelling and Heart Failure. Am. J. Nephrol..

[20] Muller D.N., Bohlender J., Hilgers K.F., Dragun D., Costerousse O., Ménard J., Luft F. (1997). Vascular Angiotensin-Converting Enzyme Expression Regulates Local Angiotensin II. Hypertension.

[21] Müller D., Luft F. (1998). The renin-angiotensin system in the vessel wall. Basic Res. Cardiol..

[22] Navar L.G., Mitchell K.D., Harrison-Bernard L.M., Kobori H., Nishiyama A. (2001). Review: Intrarenal angiotensin II levels in normal and hypertensive states. J. Renin-Angiotensin-Aldosterone Syst..

[23] Xiao F., Burns K.D. (2017). Measurement of Angiotensin Converting Enzyme 2 Activity in Biological Fluid (ACE2). Methods Mol. Biol..

[24] Yamaleyeva L.M., Gilliam-Davis S., Almeida I., Brosnihan K.B., Lindsey S.H., Chappell M.C. (2012). Differential regulation of circulating and renal ACE2 and ACE in hypertensive mRen2.Lewis rats with early-onset diabetes. Am. J. Physiol. Physiol..

[25] Yu H.-R., Tain Y.-L., Tiao M.-M., Chen C.-C., Sheen J.-M., Lin I.-C., Li S.-W., Tsai C.-C., Lin Y.-J., Hsieh K.-S. (2018). Prenatal dexamethasone and postnatal high-fat diet have a synergistic effect of elevating blood pressure through a distinct programming mechanism of systemic and adipose renin–angiotensin systems. Lipids Health Dis..

[26] Paul M., Mehr A.P., Kreutz R. (2006). Physiology of Local Renin-Angiotensin Systems. Physiol. Rev..

[27] Vehaskari V.M., Stewart T., Lafont D., Soyez C., Seth D., Manning J. (2004). Kidney angiotensin and angiotensin receptor expression in prenatally programmed hypertension. Am. J. Physiol. Physiol..

[28] Pladys P., Lahaie I., Cambonie G., Thibault G., Lê N.L.O., Abran D., Nuyt A.M. (2004). Role of Brain and Peripheral Angiotensin II in Hypertension and Altered Arterial Baroreflex Programmed during Fetal Life in Rat. Pediatr. Res..

[29] Yzydorczyk C., Gobeil F., Cambonie G., Lahaie I., Lê N.L.O., Samarani S., Ahmad A., Lavoie J.C., Oligny L.L., Pladys P. (2006). Exaggerated vasomotor response to ANG II in rats with fetal programming of hypertension associated with exposure to a low-protein diet during gestation. Am. J. Physiol. Integr. Comp. Physiol..

[30] Mao C., Zhang H., Xiao D., Zhu L., Ding Y., Zhang Y., Wu L., Xu Z., Zhang L. (2008). Perinatal nicotine exposure alters AT1 and AT2 receptor expression pattern in the brain of fetal and offspring rats. Brain Res..

[31] Goyal R., Leitzke A., Goyal D., Gheorghe C.P., Longo L.D. (2011). Antenatal maternal hypoxic stress: Adaptations in fetal lung Renin-Angiotensin system. Reprod. Sci..

[32] Mao C., Hou J., Ge J., Hu Y., Ding Y., Zhou Y., Zhang H., Xu Z., Zhang L. (2010). Changes of renal AT1/AT2 receptors and structures in ovine fetuses following exposure to long-term hypoxia. Am. J. Nephrol..

[33] Vieira-Rocha M.S., Sousa J.B., Rodriguez-Rodriguez P., Morato M., Arribas S.M., Diniz C. (2020). Insights into sympathetic nervous system and GPCR interplay in fetal programming of hypertension: A bridge for new pharmacological strategies. Drug Discov. Today.

[34] Mesquita F.F., Gontijo J.A.R., Boer P.A. (2009). Expression of renin-angiotensin system signalling compounds in maternal protein-restricted rats: Effect on renal sodium excretion and blood pressure. Nephrol. Dial. Transplant..

[35] Tsukuda K., Mogi M., Iwanami J., Min L.-J., Jing F., Ohshima K., Horiuchi M. (2012). Influence of angiotensin II type 1 receptor-associated protein on prenatal development and adult hypertension after maternal dietary protein restriction during pregnancy. J. Am. Soc. Hypertens..

[36] Gwathmey T.M., Shaltout H.A., Rose J.C., Diz D.I., Chappell M.C. (2011). Glucocorticoid-Induced Fetal Programming Alters the Functional Complement of Angiotensin Receptor Subtypes Within the Kidney. Hypertension.

[37] Marshall A.C., Shaltout H.A., Nautiyal M., Rose J.C., Chappell M.C., Diz D.I. (2013). Fetal betamethasone exposure attenuates angiotensin-(1-7)-Mas receptor expression in the dorsal medulla of adult sheep. Peptides.

[38] Sousa J., Vieira-Rocha M.S., Sá C., Ferreirinha F., Correia-De-Sá P., Fresco P., Diniz C. (2014). Lack of Endogenous Adenosine Tonus on Sympathetic Neurotransmission in Spontaneously Hypertensive Rat Mesenteric Artery. PLoS ONE.

[39] Sousa J.B., Vieira-Rocha M.S., Arribas S.M., Gonzalez-Garcia M.C., Fresco P., Diniz C. (2015). Endothelial and Neuronal Nitric Oxide Activate Distinct Pathways on Sympathetic Neurotransmission in Rat Tail and Mesenteric Arteries. PLoS ONE.

[40] Stenmark K.R., Yeager M.E., El Kasmi K.C., Nozik-Grayck E., Gerasimovskaya E.V., Li M., Riddle S.R., Frid M.G. (2013). The Adventitia: Essential Regulator of Vascular Wall Structure and Function. Annu. Rev. Physiol..

[41] Csányi G., Taylor W.R., Pagano P.J. (2009). NOX and inflammation in the vascular adventitia. Free Radic. Biol. Med..

[42] Cat A.N.D., Montezano A., Burger D., Touyz R.M. (2013). Angiotensin II, NADPH Oxidase, and Redox Signaling in the Vasculature. Antioxid. Redox Signal..

[43] Liu J., Yang F., Yang X.-P., Jankowski M., Pagano P.J. (2003). NAD(P)H Oxidase Mediates Angiotensin II–Induced Vascular Macrophage Infiltration and Medial Hypertrophy. Arter. Thromb. Vasc. Biol..

[44] Ushio-Fukai M., Zafari A.M., Fukui T., Ishizaka N., Griendling K.K. (1996). p22phox is a critical component of the superoxide-generating NADH/NADPH oxidase system and regulates angiotensin II-induced hypertrophy in vascular smooth muscle cells. J. Biol. Chem..

[45] Paravicini T.M., Touyz R.M. (2006). Redox signaling in hypertension. Cardiovasc. Res..

[46] Condezo-Hoyos L., Arribas S.M., Abderrahim F., Somoza B., Gil-Ortega M., Díaz-Gil J.J., Conde M.V., Susin C., González M.C. (2012). Liver growth factor treatment reverses vascular and plasmatic oxidative stress in spontaneously hypertensive rats. J. Hypertens..

[47] Tinajero M.G., Gotlieb A.I. (2020). Recent Developments in Vascular Adventitial Pathobiology. Am. J. Pathol..

[48] Sousa J., Diniz C., Lenasi H. (2018). Vascular Sympathetic Neurotransmission and Endothelial Dysfunction. Endothelial Dysfunction—Old Concepts and New Challenges.

[49] Somoza B., González M.C., González J.M., Abderrahim F., Arribas S.M., Fernández-Alfonso M.S. (2005). Modulatory role of the adventitia on noradrenaline and angiotensin II responses role of endothelium and AT2 receptors. Cardiovasc. Res..

[50] Rodríguez P.R., de Pablo A.L.L., Condezo-Hoyos L., Martín-Cabrejas M.A., Aguilera Y., Ruiz-Hurtado G., Gutierrez-Arzapalo P.Y., Cortijo D.R., Fernandez-Alfonso M.S., Gonzalez-Garcia M.C. (2015). Fetal undernutrition is associated with perinatal sex-dependent alterations in oxidative status. J. Nutr. Biochem..

[51] Griendling K.K., Camargo L.L., Rios F.J., Alves-Lopes R., Touyz R.M. (2021). Oxidative Stress and Hypertension. Circ. Res..

[52] Guzik T.J., Touyz R.M. (2017). Oxidative Stress, Inflammation, and Vascular Aging in Hypertension. Hypertension.

[53] Pinheiro L.C., Oliveira-Paula G.H. (2020). Sources and Effects of Oxidative Stress in Hypertension. Curr. Hypertens. Rev..

[54] Conde M.V., Gonzalez M.C., Quintana-Villamandos B., Abderrahim F., Briones A.M., Condezo-Hoyos L., Regadera J., Susin C., de Diego J.J.G., Delgado-Baeza E. (2011). Liver growth factor treatment restores cell-extracellular matrix balance in resistance arteries and improves left ventricular hypertrophy in SHR. Am. J. Physiol. Circ. Physiol..

[55] González M.C., Arribas S.M., Molero F., Fernández-Alfonso M.S. (2001). Effect of removal of adventitia on vascular smooth muscle contraction and relaxation. Am. J. Physiol. Circ. Physiol..

[56] Masi S., Uliana M., Virdis A. (2019). Angiotensin II and vascular damage in hypertension: Role of oxidative stress and sympathetic activation. Vasc. Pharmacol..

[57] Schiffrin E.L. (2012). Vascular remodeling in hypertension: Mechanisms and treatment. Hypertension.

[58] Forrester S.J., Booz G.W., Sigmund C.D., Coffman T.M., Kawai T., Rizzo V., Scalia R., Eguchi S. (2018). Angiotensin II Signal Transduction: An Update on Mechanisms of Physiology and Pathophysiology. Physiol. Rev..

[59] Mathiassen O.N., Buus N.H., Sihm I., Thybo N.K., Mørn B., Schroeder A.P., Thygesen K., Aalkjaer C., Lederballe O., Mulvany M.J. (2007). Small artery structure is an independent predictor of cardiovascular events in essential hypertension. J. Hypertens..

[60] Rizzoni D., Porteri E., Boari G.E., De Ciuceis C., Sleiman I., Muiesan M.L., Castellano M., Miclini M., Agabiti-Rosei E. (2003). Prognostic Significance of Small-Artery Structure in Hypertension. Circulation.

[61] Lehoux S. (2016). Adventures in the Adventitia. Hypertension.

[62] Ehret G.B., Munroe P.B., Rice K.M., Bochud M., Johnson A.D., Chasman D.I., Smith A.V., Tobin M.D., Verwoert G.C., Hwang S.-J. (2011). Genetic variants in novel pathways influence blood pressure and cardiovascular disease risk. Nature.

[63] Percie du Sert N., Hurst V., Ahluwalia A., Alam S., Avey M.T., Baker M., Browne W.J., Clark A., Cuthill I.C., Howells D.W. (2020). The ARRIVE guidelines 2.0: Updated guidelines for reporting animal research. PLoS Biol..

[64] Muñoz-Valverde D., Rodríguez P.R., Gutierrez-Arzapalo P.Y., De Pablo A.L.L., González M.C., López-Giménez R., Somoza B., Arribas S.M. (2015). Effect of Fetal Undernutrition and Postnatal Overfeeding on Rat Adipose Tissue and Organ Growth at Early Stages of Postnatal Development. Physiol. Res..

[65] Hawkins C.L., Morgan P.E., Davies M.J. (2009). Quantification of protein modification by oxidants. Free Radic. Biol. Med..

[66] Ferreira-Duarte M., Rodrigues-Pinto T., Sousa T., Faria M., Rocha M., Menezes-Pinto D., Esteves-Monteiro M., Magro F., Dias-Pereira P., Duarte-Araújo M. (2021). Interaction between the Renin–Angiotensin System and Enteric Neurotransmission Contributes to Colonic Dysmotility in the TNBS-Induced Model of Colitis. Int. J. Mol. Sci..

[67] Rocha-Pereira C., Sousa J., Rocha M.S.V., Fresco P., Gonçalves J., Diniz C. (2013). Differential inhibition of noradrenaline release mediated by inhibitory A1-adenosine receptors in the mesenteric vein and artery from normotensive and hypertensive rats. Neurochem. Int..

[68] Arribas S.M., Hillier C., González C., McGrory S., Dominiczak A., McGrath J. (1997). Cellular Aspects of Vascular Remodeling in Hypertension Revealed by Confocal Microscopy. Hypertension.

[69] Sousa J.B.P.F.a.C.D., Méndez-Villas A. (2014). Imaging receptors with Laser Scanning Confocal Microscopy: Qualitative and quantitative analysis. Microscopy: Advances in Scientific Research and Education.

[70] Sabatino L., Costagli C., Lapi D., Del Seppia C., Federighi G., Balzan S., Colantuoni A., Iervasi G., Scuri R. (2018). Renin-Angiotensin System Responds to Prolonged Hypotensive Effect Induced by Mandibular Extension in Spontaneously Hypertensive Rats. Front. Physiol..

[71] Livak K.J., Schmittgen T.D. (2001). Analysis of relative gene expression data using real-time quantitative PCR and the 2(-Delta Delta C(T)) method. Methods.

[72] Vandesompele J., De Preter K., Pattyn F., Poppe B., Van Roy N., De Paepe A., Speleman F. (2002). Accurate normalization of real-time quantitative RT-PCR data by geometric averaging of multiple internal control genes. Genome Biol..

[73] Schindelin J., Arganda-Carreras I., Frise E., Kaynig V., Longair M., Pietzsch T., Preibish S., Rueden C., Saalfeld S., Schmid B. (2012). Fiji: An open-source platform for biological-image analysis. Nat. Methods.

